# Supplementary material for: PLGA scaffold carrying icariin to inhibit the progression of osteoarthritis in rabbits
Source: R Soc Open Sci. 2019 Apr 10;6(4):181877. doi: 10.1098/rsos.181877 (PMC6502375; doi:10.1098/rsos.181877)
Supplement: ethical approval [file rsos181877supp1.pdf]

伦理审查批件

审查号：CCZYFYLL2017 审字- 057

审查日期	2017 年 11 月 15 日		
审查地点	长春市工农大路 1478 号		
临床研究批文	不适用		
临床研究项目	淫羊藿对骨性关节炎软骨细胞分化及凋亡的作用研究		
审查文件	■初始审查申请表　■临床研究方案（版本号：1.0）　■知情同意书（版本号：1.0）<br>■项目任务书　■主要研究者履历		
申办者/CRO	长春中医药大学附属医院		
临床研究单位	长春中医药大学附属医院骨科中心		
主要研究者	李绍军		
伦理审查方式	■会议审查　□快速审查		
审查人员	郝东明，王秀阁，邓悦，李新建，冷炎，姜丽红，夏淑范，韩祎，刘怀，李琳，杨铁峥，连树林		
审查意见	根据中华人民共和国国家食品药品监督管理局 2003 年颁布实施的《药物临床试验质量管理规范》以及《赫尔辛基宣言》和国际医学科学组织委员会颁布的《人体生物医学研究国际道德指南》的伦理原则，经本伦理委员会审查，同意在长春中医药大学附属医院进行“淫羊藿对骨性关节炎软骨细胞分化及凋亡的作用研究（方案版本号：1.0）”临床试验。<br>本批件将在本中心机构及其伦理委员会备案。如果对方案在本机构的可行性（包括研究者的资格与经验、设备与条件等）有不同意见，请及时与本伦理委员会联系。<br>本审查自签发日期有效期 2 年，研究负责人必须严格使用经审查同意的知情同意书文本和研究方案。如伦理审查批件失效时不能完成所有的临床研究（包括统计分析），请在本批件失效前一个月，递交跟踪审查申请。请于 2018 年 11 月 16 日前 1 个月提交跟踪审查；如研究结束并在审查有效期内，请向本伦理委员会提交研究结题报告；暂停/提前终止临床研究，请及时通知伦理委员会；如发生严重不良事件以及影响研究风险受益比的非预期不良事件，应及时报告本伦理委员会；临床研究方案、知情同意书的任何修改，包括主要研究者的更换等，需递交研究方案修改申请表，经伦理委员会重新审查，获得批准后执行；发现影响受试者参加研究意愿的违反方案情况应及时报告。		
批件有效期	2017 年 11 月 16 日～2019 年 11 月 16 日	联系电话	高宏伟：0431-86177876

主任委员签字：

伦理委员会盖章

日期：2017 年 11 月 16 日
